# Supplementary material for: Proteus: a random forest classifier to predict disorder-to-order transitioning binding regions in intrinsically disordered proteins
Source: J Comput Aided Mol Des. 2017 Apr 1;31(5):453–66. doi: 10.1007/s10822-017-0020-y (PMC5429364; doi:10.1007/s10822-017-0020-y)

Supporting Figures

Fig.S1. The evaluation scores as a function of maximum tree depth for the classifier.

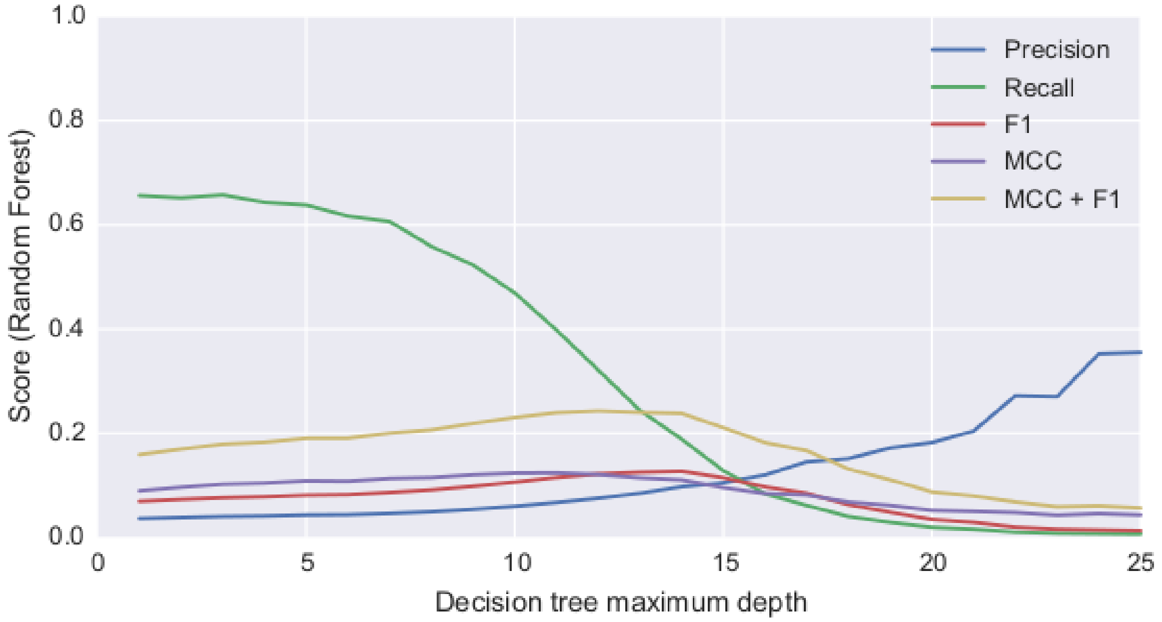

Fig.S2. The evaluation scores as a function of the number of decision trees.

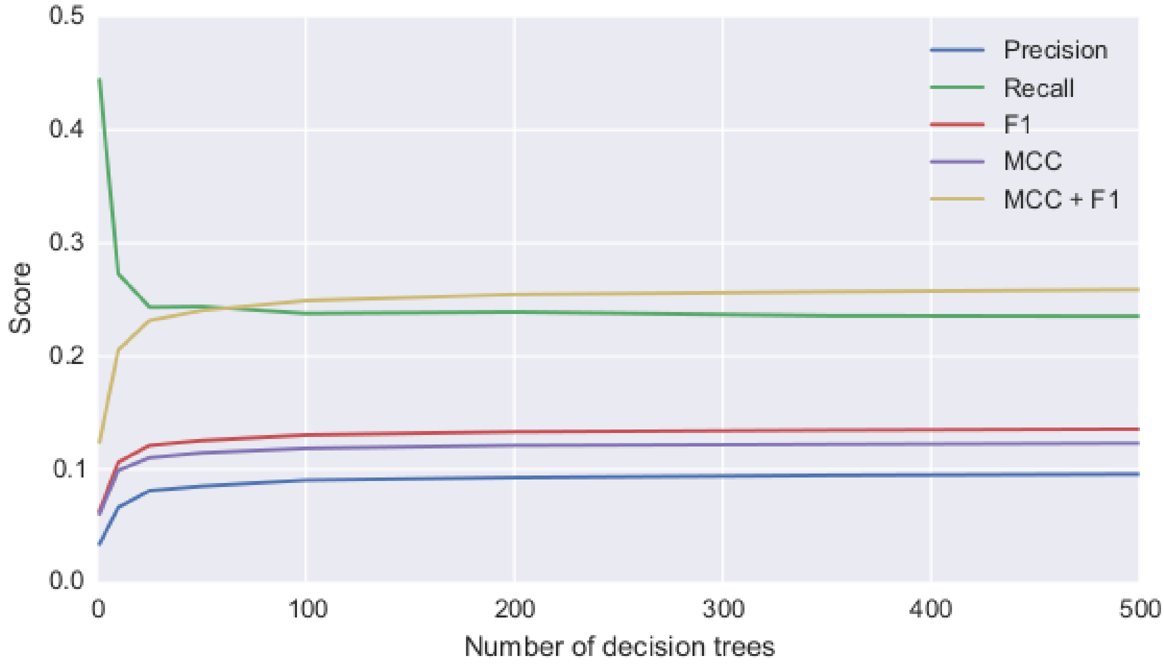

Fig.S3. The evaluation scores as a function of the probability cut-off (P_{cut}) referred to as 'Threshold' in the X-axis.

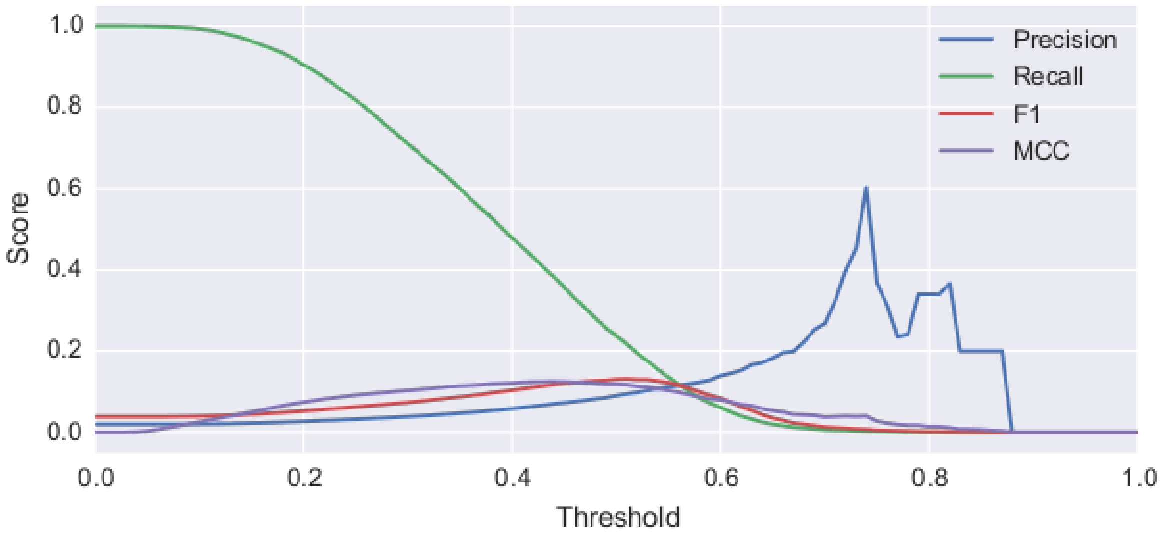

Fig.S4. The disordered region around residue 50 in protein 1E91 B. The length of the disordered region is highlighted in green.

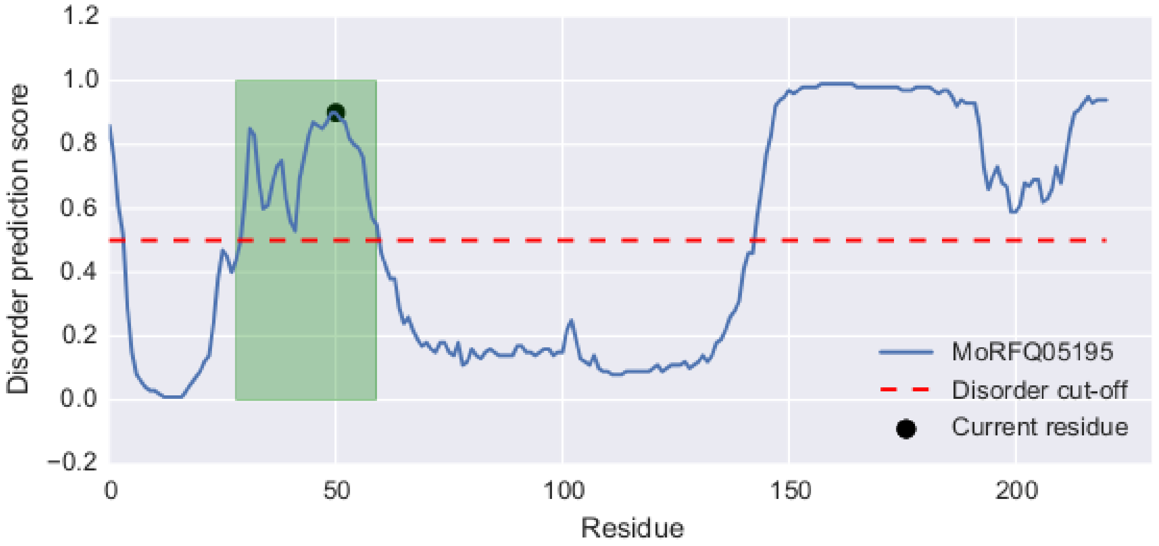

Fig.S5. The evaluation scores as a function of window size.

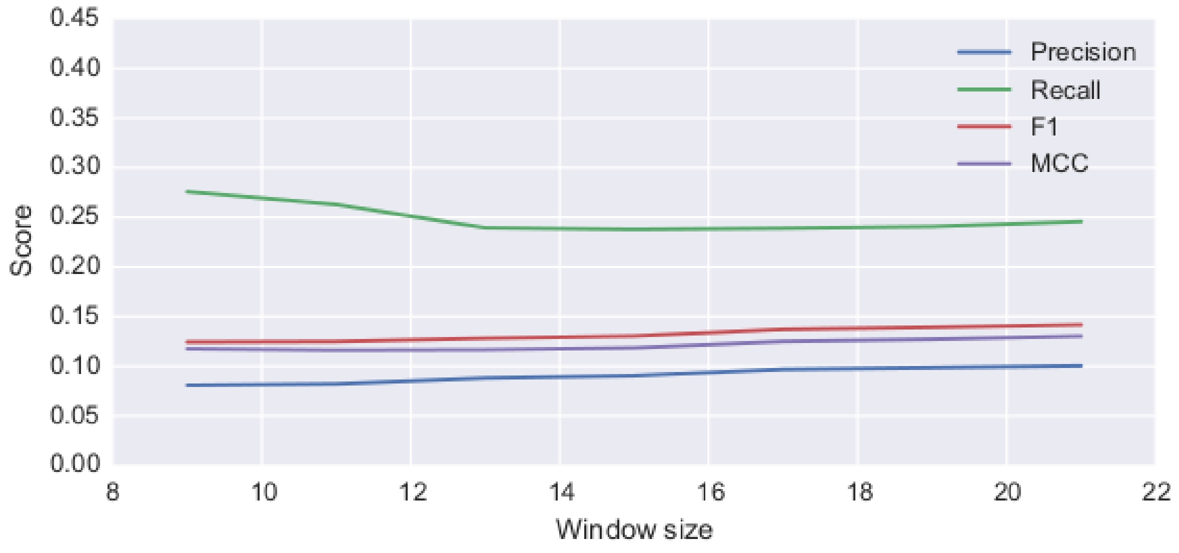

Fig.S6. The 20 best combinations of feature groups as measured by the MCC score. The numbers refer to the feature groups presented in Materials and Methods and summarized in Table 1. Feature Group 1: Sequence Profiles; 2: Amino Acid Conservation; 3: Amino Acid Concentration; 4: Amino Acid Properties; 5: Secondary Structure; 6: Predicted Disorder; 7: Disorder Topography

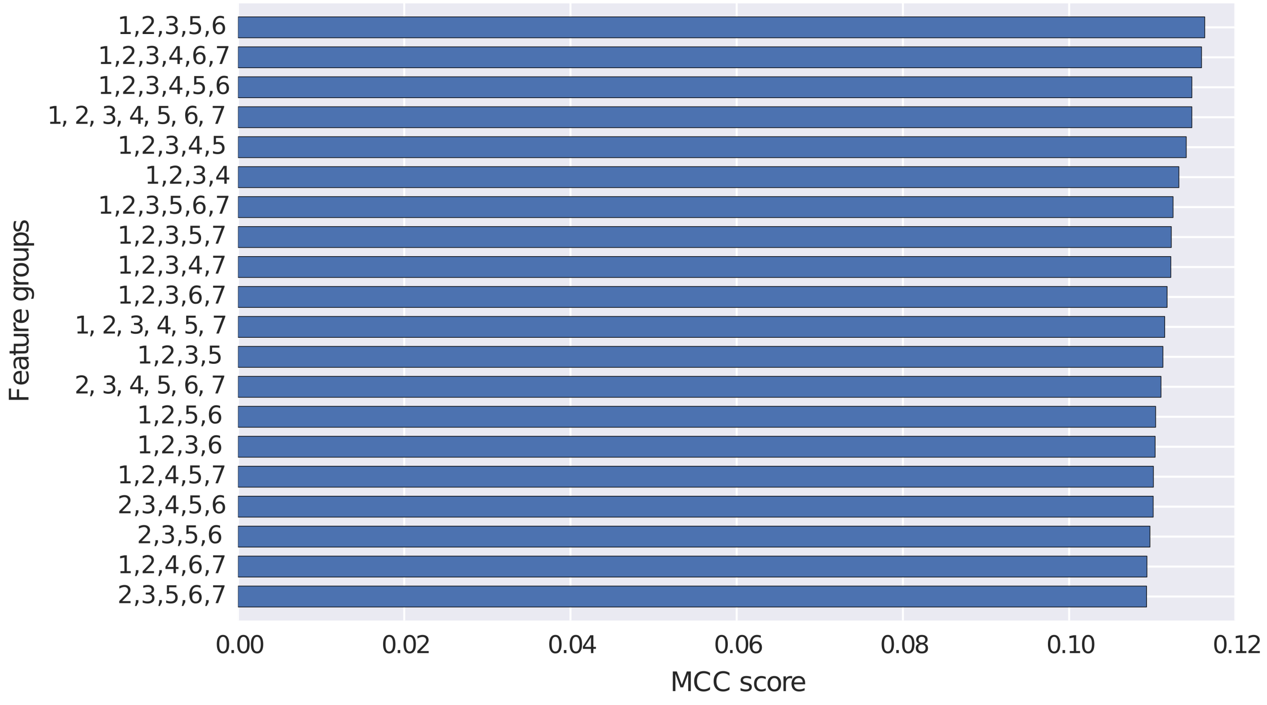

Fig.S7. The 20 best combinations of feature groups as measured by the F1 score. The numbers refer to the feature groups presented in the Materials and Methods and summarized in Table 1. Feature Group 1: Sequence Profiles; 2: Amino Acid Conservation; 3: Amino Acid Concentration; 4: Amino Acid Properties; 5: Secondary Structure; 6: Predicted Disorder; 7: Disorder Topography.

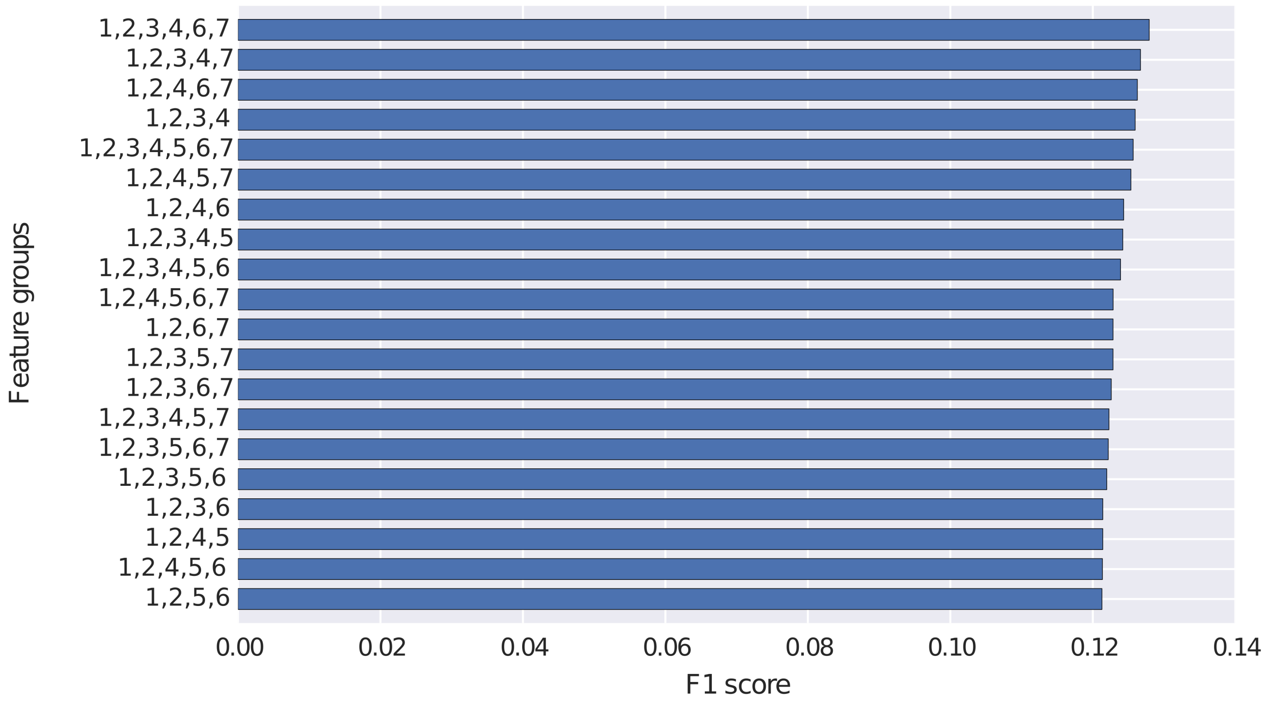

Fig.S8. Comparison of recall vs. precision curves for Proteus and other classifiers. All methods were tested on the same independent validation set of 9 proteins. AUC stands for Area Under the Curve which were calculated using the Trapezoidal numerical integration (trapz) function of MATLAB. The random baseline (dashed black line) corresponds to a purely random classifier.

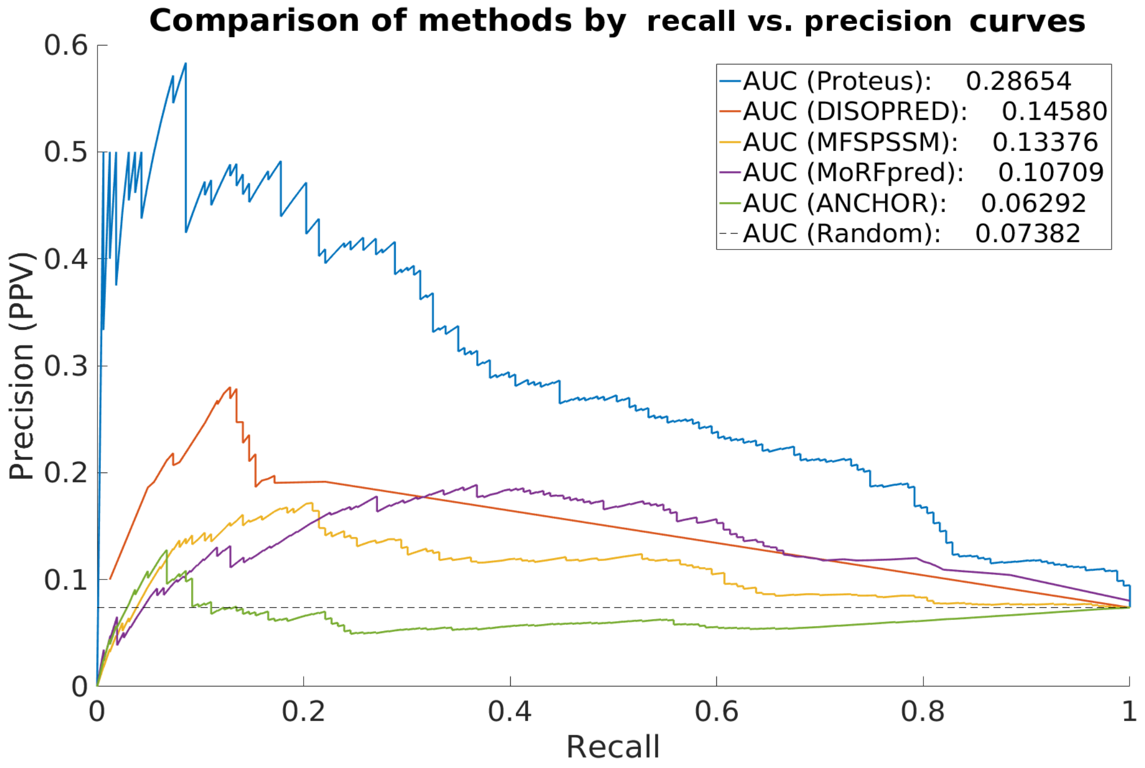

Supplement: Supplementary file 1 — Supplementary material 1 (PDF 742 KB) [file 10822_2017_20_MOESM1_ESM.pdf]
